# Supplementary material for: Training, Attitudes, and Practice (TAP) among healthcare professionals in the Nelson Mandela Bay municipality, South Africa: A health promotion and disease prevention perspective
Source: PLoS One. 2021 Nov 24;16(11):e0259884. doi: 10.1371/journal.pone.0259884 (PMC8612580; doi:10.1371/journal.pone.0259884)
Supplement: S1 File — (DOCX) [file pone.0259884.s001.docx]

**TAP Questionnaire**

Section one

| S/N | Questions | Responses | |
| --- | --- | --- | --- |
| 1 | Sex | Male | Female |
| 2 | What is your healthcare (HC) facility level? | Primary HC facility |  |
|  |  | Secondary HC facility |  |
|  |  | Tertiary HC facility |  |
| 3 | Are you a registered HC professional in South Africa? | Yes | No |
| 4 | If yes, in what profession were you registered? (please tick the appropriate one) | Medical doctor |  |
|  |  | Registered Nurse |  |
|  |  | Dietician |  |
|  |  | Physiotherapist |  |
|  |  | Speech Therapist |  |
|  |  | Occupational Therapist |  |
|  |  | Social worker |  |

Section two – Training infrastructure for HP and DP (Kindly tick your preferred option)

| S/N | Questions | Responses |  |
| --- | --- | --- | --- |
| 5 | Is there a coordinated HP training for staff? | Yes | No |
| 6 | Have you ever participated in any HP training? | Yes | No |
| 7 | If yes, what benefits do think were derived from the training | a. Improved knowledge |  |
|  |  | b. Improved skills |  |
|  |  | c. Enhanced confidence |  |
|  |  | d. positive staff behavior, attributions and emotional responses |  |
|  |  | e. Enhanced staff satisfaction |  |
|  |  | f. increased support to patients |  |
|  |  | g. Enhanced staff retention |  |
|  |  | h. No added benefit |  |

| 8 | Is there a continuing  professional development for health promotion in your facility | Yes | No | I don’t know |
| --- | --- | --- | --- | --- |

Section 3- Attitudes towards HP/DP

| S/N | Questions |  |  |  |  |  |
| --- | --- | --- | --- | --- | --- | --- |
|  |  | Strongly Disagree | Disagree | Neutral | Agree | Strongly Agree |
| 9 | HCW should model good health behavior in order to give HP advice |  |  |  |  |  |
| 10 | HCW should be encouraged to engage in HP as part of government policy and healthcare services |  |  |  |  |  |
| 11 | Health promotion is a waste of time |  |  |  |  |  |
| 12 | Patients who deliberately engage in an unhealthy lifestyle will not benefit from health promotion |  |  |  |  |  |
| 13 | Health education, advise and counseling from HCW could positively enhance patients’ health |  |  |  |  |  |
| 14 | I do not have time to implement health promotion |  |  |  |  |  |
| 15 | Patients do not want health education from HCW |  |  |  |  |  |

| 16 | Do you think HCW should participate in HP? | Yes | No |
| --- | --- | --- | --- |
