# Supplementary material for: Training, Attitudes, and Practice (TAP) among healthcare professionals in the Nelson Mandela Bay municipality, South Africa: A health promotion and disease prevention perspective
Source: PLoS One. 2021 Nov 24;16(11):e0259884. doi: 10.1371/journal.pone.0259884 (PMC8612580; doi:10.1371/journal.pone.0259884)
Supplement: Attachment — Submitted filename: Response to reviewers comments 151021.docx [file pone.0259884.s003.docx]

**Rebuttal Table**

**Reviewer 1**

| **Comments** | **Responses** | **Page and line numbers where changes have been made** |
| --- | --- | --- |
| To improve an international audience understanding of content, add definitions for primary, secondary, and tertiary facilities. | Thank you for pointing this out and we have defined the terms as follows:  A primary health care facility refers to facilities where ambulatory or first-contact personal health care services are provided (26) and in this context includes the Primary Healthcare Centres (PHC), community clinics, Comprehensive Health Centers (CHC), and Midwife Obstetrics units (MOU). A secondary-level hospital refers to a healthcare facility that is highly differentiated by function with five to ten clinical specialties (27). This level of facility includes the Regional Hospitals, Provincial Hospitals, and General Hospitals. The capacity ranges between 200 and 800 beds. Tertiary-level hospitals on the other hand are hospitals with highly specialized professionals and facilities such as cardiology, intensive care unit, and specialized imaging infrastructures among others (27). The clinical services of the tertiary healthcare facilities are highly differentiated by function and have capacity of between 300 and 1500 beds. This level of hospitals includes National Hospitals, Central Hospitals, and Academic, or teaching or University Hospitals. | Page 4, lines 138-149 |
| Replace to “our knowledge: with “based on the literature review for this project, this is only…” | As suggested by the reviewer, this change has been done | Pages 3 lines 97-99. |

**Reviewer 2**

| **Comments** | **Responses** | **Page and line numbers where changes have been made** |
| --- | --- | --- |
| 1. Why was this municipality chosen? How many HCP work in this municipality overall? | The choice for NMBM and number of HCPs in the municipality have been included and cited accordingly.  The NMBM is the largest City in the Eastern Cape Province and a major economic player both for the province and South Africa as a whole (24). Like the rest of South Africa, the municipality’s healthcare system has all three levels of care comprising primary, secondary and tertiary in place. Despite availability of inexpensive measures such as dietary counselling, physical activity, and handwashing for HP and DP, HCPs do not have structured training and knowledge to effectively implement the measures (13). Furthermore, there is limited evidence on the HP and DP training and attitudes of HCPs in relation to HP and DP especially in resource constrained settings (25,26).  The municipality has an estimated 3500 healthcare professionals (27) serving a population of about 1.24 million | Page 3, lines 91-97  Page 3, lines 116-117 |
| 2. How were the 500 HCP chosen to participate? Convenience sample? What was the response rate? | We have justified the sample size and inserted the response rate accordingly.  This quantitative cross-sectional study was carried out among 520 HCPs that were randomly sampled from 23 hospitals in the NMBM. Only 501 HCPs completed the survey. Of the 501 respondents, six HCPs did not include their professions and were excluded from analysis. A total of 495 HCPs were included in the final analysis. An initial sample size of 384 was reached using a confidence level of 95% and an error margin of 5%. This sample size was further adjusted by 25% to 480 to account for non-responding HCPs.  The response rate for this study was 95% (n=495). | Pages 3-4, lines 120-125  Page 5, line 178 |
| 3. Typically any results are reported as x/y (z%) | We agree with this suggestion and have included tables 1, 2 and 4 to address this. | Table 1- Page 5, lines 192-193  Table 2- Page 8, line 206  Table 4 - Page 13, lines 232-234 |
| 4. How did the questions decide on the survey questions to include? Was this based on any prior validated survey? Was the survey pilot tested for face or content validity? | We have explained how the questions were decided as follows:  A standardized questionnaire adapted from a previously validated study (23) was developed to determine the influence of HCPs’ training and attitude on their practice of HP and DP. The questionnaire was further subjected to a pilot test. | Page 4, lines 133-135  Page 4: Lines 158-159 |
| 5. "The risk of coordinated HP training for staff for medical doctors" wording is awkward; would rewrite. | Revised accordingly. “The risk of coordinated HP training for medical doctors” | Page 10, line 211 |
| 6. It would be helpful for Table 1 to list the demographics | Thank you for pointing this out. Although we agree that this is an important consideration, we have summarized the socio-demographic characteristics in page 5 to keep our tables concise. |  |
| 7. The tables could be laid out more clearly and list the overall % of each group who agreed with the various statements rather than limited to Coefficients. | We agree with this suggestion and have included tables 1,2 and 4 to address this. | Table 1- Page 5, lines 192-193  Table 2- Page 8, line 206  Table 4 - Page 13, lines 232-234 |
